# Short-term paleogeographic reorganizations and climate events shaped diversification of North American freshwater gastropods over deep time

**DOI:** 10.1038/s41598-022-19759-4

**Published:** 2022-09-16

**Authors:** Thomas A. Neubauer, Mathias Harzhauser, Joseph H. Hartman, Daniele Silvestro, Christopher R. Scotese, Alexander Czaja, Geerat J. Vermeij, Thomas Wilke

**Affiliations:** 1 Department of Animal Ecology and Systematics, Justus Liebig University, 35392 Giessen, Germany; 2 SNSB–Bavarian State Collection for Paleontology and Geology, Richard-Wagner-Straße 10, 80333 Munich, Germany; 3 Naturalis Biodiversity Center, 2333 CR Leiden, The Netherlands; 4 Geological-Paleontological Department, Natural History Museum Vienna, 1010 Vienna, Austria; 5 University of North Dakota, Harold Hamm School of Geology and Geological Engineering, Grand Forks, ND 58202 USA; 6 Department of Biology, University of Fribourg, 1700 Fribourg, Switzerland; 7 Gothenburg Global Biodiversity Centre, University of Gothenburg, 413 19 Gothenburg, Sweden; 8 Department of Biological and Environmental Sciences, University of Gothenburg, 405 30 Gothenburg, Sweden; 9 Swiss Institute of Bioinformatics, 1015 Lausanne, Switzerland; 10 Department of Earth and Planetary Sciences, Northwestern University, Evanston, IL 60208 USA; 11 Facultad de Ciencias Biológicas, Universidad Juárez del Estado de Durango, Fraccionamiento Filadelfia, 35010 Gómez Palacio, Durango Mexico; 12 Department of Earth and Planetary Science, University of California, Davis, CA 95616 USA

**Keywords:** Palaeontology, Speciation

## Abstract

What controls species diversity and diversification is one of the major questions in evolutionary biology and paleontology. Previous studies have addressed this issue based on various plant and animal groups, geographic regions, and time intervals. However, as most previous research focused on terrestrial or marine ecosystems, our understanding of the controls on diversification of biota (and particularly invertebrates) in freshwater environments in deep time is still limited. Here, we infer diversification rates of North American freshwater gastropods from the Late Triassic to the Pleistocene and explore potential links between shifts in speciation and extinction and major changes in paleogeography, climate, and biotic interactions. We found that variation in the speciation rate is best explained by changes in continental fragmentation, with rate shifts coinciding with major paleogeographic reorganizations in the Mesozoic, in particular the retreat of the Sundance Sea and subsequent development of the Bighorn wetland and the advance of the Western Interior Seaway. Climatic events in the Cenozoic (Middle Eocene Climate Optimum, Miocene Climate Optimum) variably coincide with shifts in speciation and extinction as well, but no significant long-term association could be detected. Similarly, no influence of diversity dependence was found across the entire time frame of ~ 214 Myr. Our results indicate that short-term climatic events and paleogeographic changes are relevant to the diversification of continental freshwater biota, while long-term trends have limited effect.

## Introduction

A crucial question in evolutionary biology and paleobiology is what controls species diversification, i.e., speciation and extinction, over geological time^1–8^. Previous studies presented a variety of hypotheses, suggesting that the controls on speciation and extinction differ greatly among paleogeographic regions, geologic time intervals, and taxonomic groups. Several abiotic factors have been hypothesized to impact species diversification including climate, sea-level fluctuations, environmental factors, and continental fragmentation^3,9–14^. A biotic factor found to be relevant in many studies is diversity dependence, whereby greater species richness slows down diversification, e.g., due to competition^2,4,5,15–18^. Several studies show a combined effect of abiotic and biotic factors on species diversification^3,12,19,20^.

The great variability in the driving forces behind diversification and in the individual contributions of biotic or abiotic factors shown in previous studies is likely owed to the chosen temporal, spatial, and environmental scales and the targeted taxonomic groups. For example, variation in the tectonic setting is a relevant control on diversification over long time spans and across continental or global scales^3,21,22^. Conversely, biotic factors such as diversity dependence are primarily regional phenomena, since species interactions happen at a local scale, while in larger systems not all members of a species group co-occur^23^. Notably though, several studies have still found support for diversity-dependent diversification on continental^2,18,24^ or even global scales^3,11,25^. Moreover, the drivers of diversification are expected to vary among marine, terrestrial, and freshwater biota given their different environmental conditions and constraints on dispersal mechanisms^26^. Hence, the roles of individual factors (or sets of factors) are best assessed in the context of spatial, temporal, and ecological heterogeneity of the studied system.

Particularly in continental settings that undergo major tectonic and environmental reorganization across geological time, where a species group would likely be biogeographically structured, we may consider biotic interactions an unlikely agent of diversification over long geological time intervals. We hypothesize that in such a system the species diversification process is controlled by abiotic factors, such as long-term climate trends and continental fragmentation^21,22^.

The North American continent and its Mesozoic–Cenozoic fossil record of freshwater gastropods provide an ideal model system to test this hypothesis. The continent has experienced substantial changes in its paleogeographic configuration and has undergone significant climatic change over this time span^27,28^. Notable paleogeographic reorganizations include the advance and retreat of major intracontinental seaways and embayments in the Mesozoic, such as the Sundance Sea and the Western Interior Seaway^29–32^.

Here, we test the potential influence of selected abiotic and biotic factors on freshwater mollusk diversification in a continental setting undergoing climatic changes and paleogeographic fragmentation, using the North American fossil record of freshwater gastropods as a model. We specifically test whether deep-time speciation and extinction dynamics can be explained by temporal variation in (i) the continent’s paleogeographic configuration, which likely affected the type, extent and connectivity of freshwater ecosystems, (ii) regional and global temperature, and (iii) species diversity (to test for diversity dependence).

## Results

**Figure 1:**
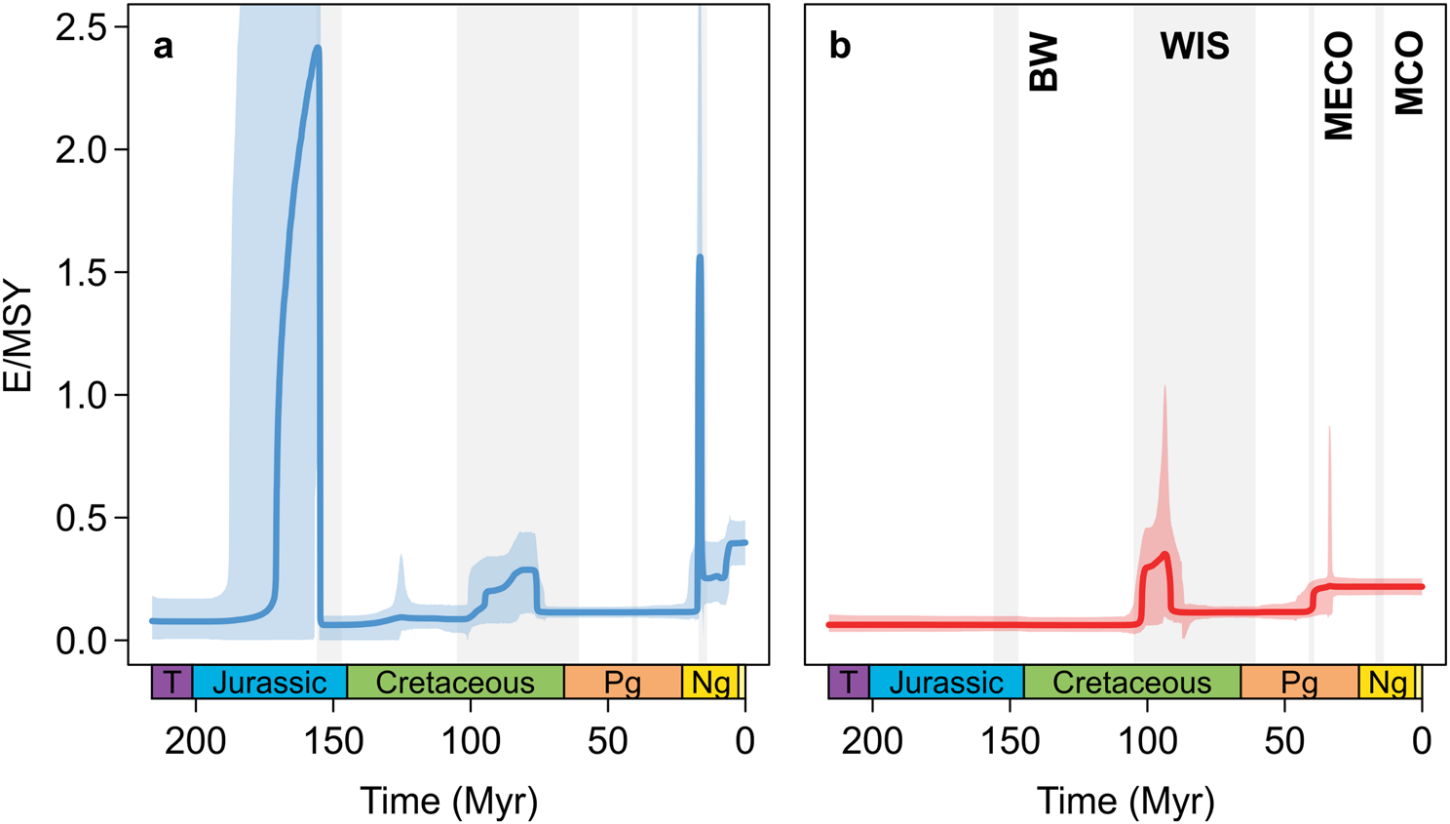
Speciation (**a**) and extinction (**b**) rates of North American freshwater gastropods from the Late Triassic to the Pleistocene (**c** 214.0–0.0117 Myr ago). Shown are the mean rates and the 95% highest posterior density quantifying the uncertainty in rates. Time intervals with major seas/wetlands and climatic events referred to in the text are marked by gray bars. The y-axis is cropped to focus on the shifts in diversification; see Fig. S2 for the complete version. BW, Bighorn wetlands; E/MSY, events per million species years; MECO, Middle Eocene Climate Optimum; MCO, Miocene Climate Optimum; WIS, Western Interior Seaway.

The North American fossil freshwater gastropod fauna comprises 606 species in 24 families ranging from the Late Triassic to the Pleistocene (c. 214.0–0.0117 Myr ago). Estimated diversification rates varied little over most of that time interval, especially the extinction rate (Fig. 1). Major peaks in the speciation rate were recorded for the Middle to Late Jurassic (although linked with a wide credible interval) and the Miocene. Elevated speciation rates were also found in the Late Cretaceous and the late Neogene (Figs. 1a, S2a). The extinction rate was elevated in the mid-Cretaceous and reached a minor plateau in the mid-Eocene (Figs. 1b, S2b).

**Figure 2:**
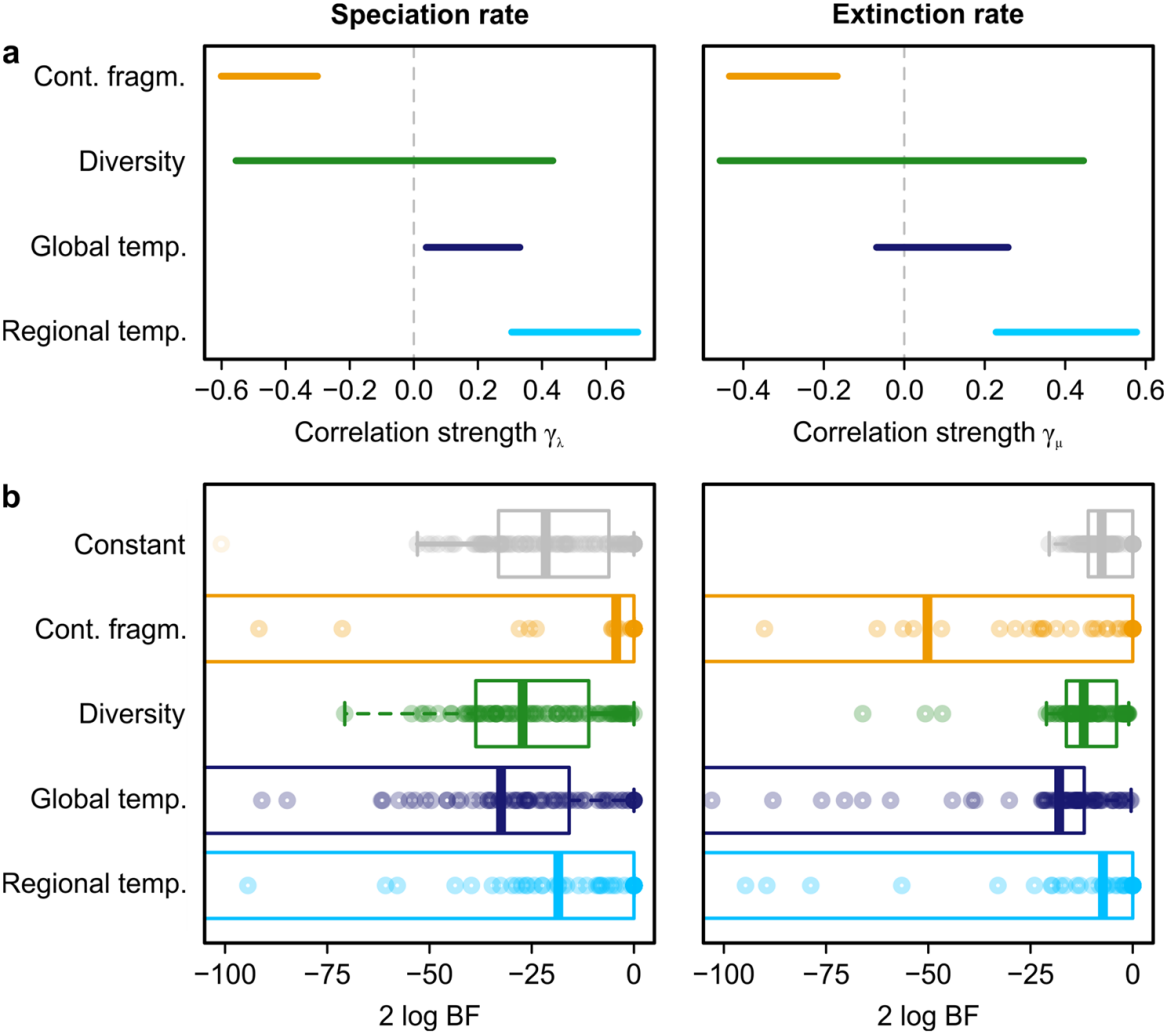
Results of the unifactorial models with speciation and extinction rates. (**a**) Regional temperature, continental fragmentation (using the Shoreline Development Index) and partly global temperature showed significant correlations with speciation and extinction rates (95% credible interval different from zero). (**b**) Bayes factors (BF) indicated that the models for SDI and regional temperature are significantly better than a constant model, but only for speciation rate. The plot is truncated at − 100 to focus on the differences in median Bayes factors; see Supplementary Table S1 for the complete data.

**Figure 3:**
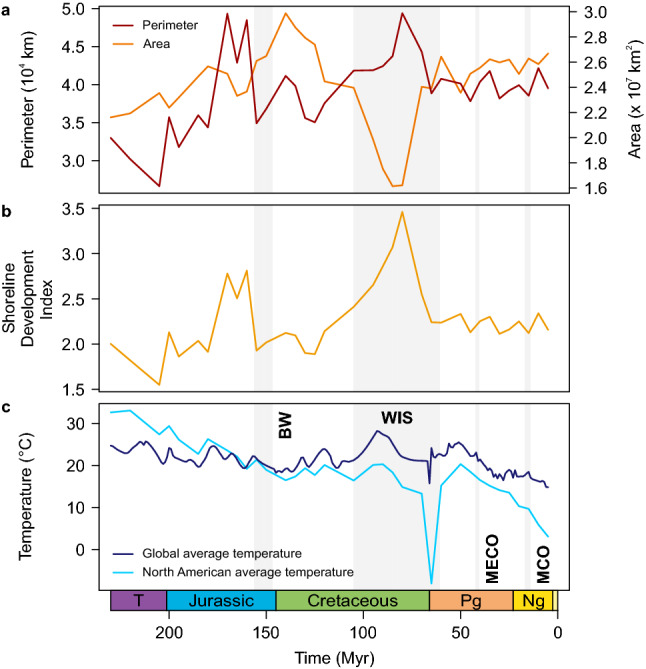
Abiotic factors used in this study. (**a**) Continental area and perimeter, (**b**) the resulting Shoreline Development Index (SDI), as well as (**c**) regional and global average temperature through geological time. Time intervals with major seas/wetlands and climatic events referred to in the text are marked by gray bars. Note that the last time bin (0–5 Myr ago) was omitted; see Methods for details. BW, Bighorn wetlands; MECO, Middle Eocene Climate Optimum; MCO, Miocene Climate Optimum; WIS, Western Interior Seaway.

The unifactorial models indicated a negative correlation with continental fragmentation, quantified using the Shoreline Development Index (SDI), and a positive correlation with regional temperature (Figs. 2a, 3). There was no significant correlation with diversity. Among all factors, the model with SDI was found to best represent the speciation rate according to Bayes factors (Fig. 2b). Also, the regional temperature model was found to explain variation in the speciation rate slightly better than the constant model (Δ2 log BF = 3.088). For the extinction rate, regional temperature had the lowest median Bayes factors, but the model was not significantly better than a constant model (Δ2 log BF = 0.303; Fig. 2b). Global temperature was positively correlated with trends in the speciation rate but not the extinction rate (Fig. 2a); in both cases, Bayes factors indicated that the models were significantly worse than a constant model (Fig. 2b).

## Discussion

Our results indicate that continental fragmentation is a main control for speciation in North American freshwater gastropods over deep time. Regional temperature variation was found to explain speciation rate only marginally better than a constant model, and none of the abiotic or biotic factors showed a significant correlation with extinction rate. However, the lack of any significant correlation is contradicted by a striking coincidence between several shifts and major paleogeographic changes and climatic events (Figs. 1, 3). Below, we discuss the correlation with individual factors, the implications for our understanding of deep-time diversification processes, as well as potential limitations of our interpretations and the use of the selected factors.

### Paleogeography: deep-time vs. short-term impact on diversification

Our models suggest that continental fragmentation is a significant control of variation in the speciation rate over long spans of geological time (Fig. 1). This result confirms findings by several other studies suggesting that changes in the paleogeographic setting influence diversification of continental faunas on a large scale^3,21,33,34^. A more fragmented continent leads to poorly connected freshwater ecosystems, which in turn directly affects biogeographic relationships and colonization and speciation processes. Also on a smaller spatiotemporal scale, variation in drainage patterns and hydrological connections plays an important role in the biogeography of freshwater gastropods^35,36^.

**Figure 4:**
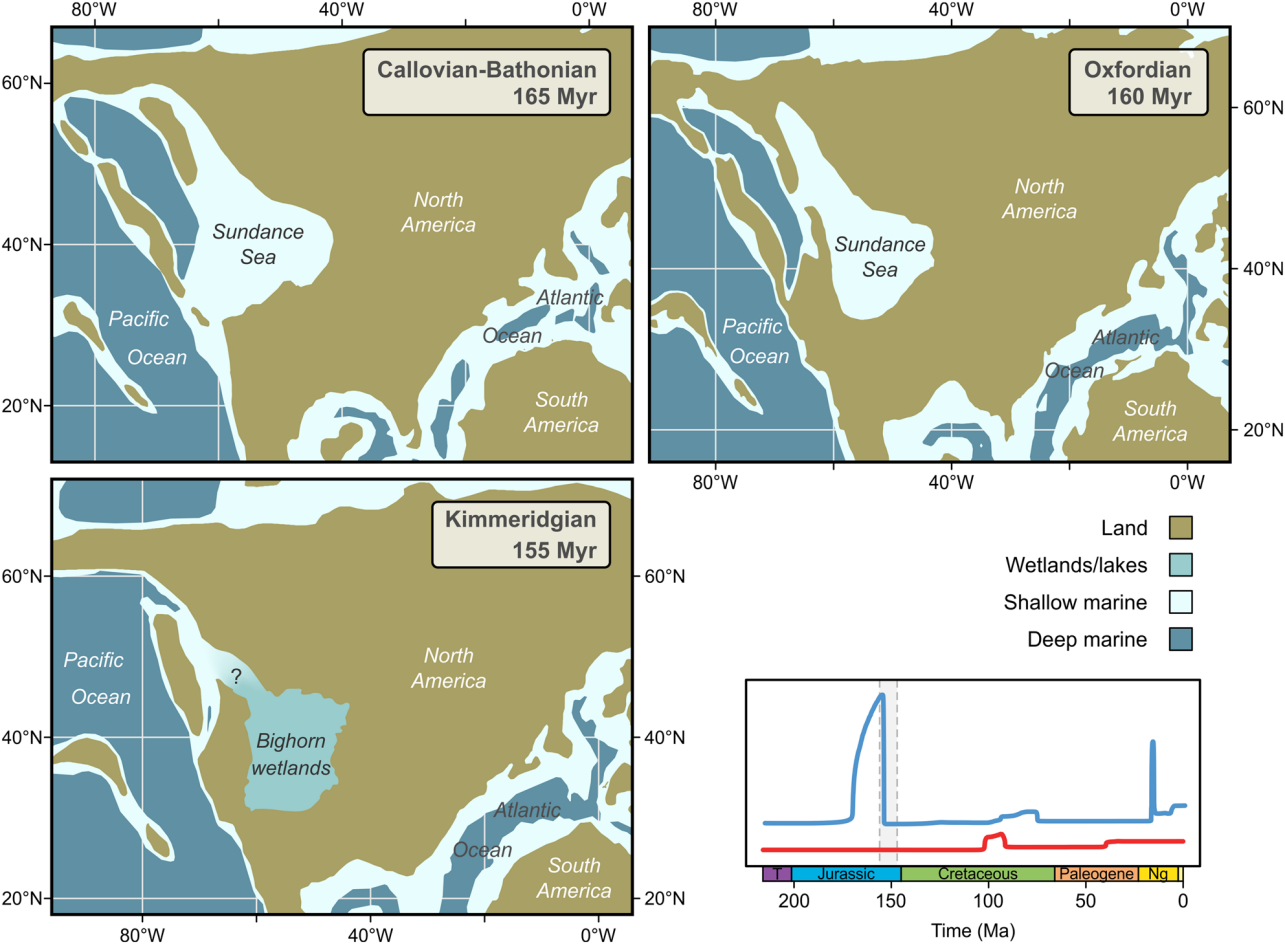
Paleogeography of western North America during the Middle to Late Jurassic, featuring the transgression and retreat of the Sundance Sea and the subsequent development of the Bighorn wetlands. The graph on the lower right indicates the temporal extent of the Bighorn wetlands^37^ and the corresponding peak in the speciation rate (blue) (see also Fig. 1). Map was generated in R v. 4.1.2 (https://www.r-project.org/) based on polygons from Ref.^27^. The extent of the Bighorn wetlands follows Ref.^72^.

In our case, shifts in the speciation rate coincide strikingly with major changes in paleogeography, more specifically with the opening and closing of intracontinental seaways during the Mesozoic (Fig. 1). The major speciation peak in the Middle to Late Jurassic matches the temporal extent of the Sundance Sea, which covered large parts of today’s northwestern United States and southwestern Canada during the Bajocian and Oxfordian stages^31,32^ (Fig. 4). The timing of the maximum peak of the speciation rate, however, slightly postdates the end of the Sundance Sea and correlates with the origin of extensive freshwater environments represented in the Morrison Formation (Kimmeridgian–Tithonian)^37^. That formation comprises a diverse set of biofacies, representing different types of freshwater habitats^37,38^, which are summarized under the term “Bighorn wetlands”^37^ (here including Lake T’oo’dichi’). A diverse gastropod fauna inhabited this ecosystem, comprising species of Viviparidae, Valvatidae, Lymnaeidae, Planorbidae, Amnicolidae, and Neritidae, amongst others^39^. We hypothesize that the sudden appearance of these new environments provided the ecological opportunities^40^ that triggered diversification and resulted in the observed boost in the speciation rate. However, the large credible interval characterizing the peak (Figs. 1a, S2) calls for caution not to overestimate the exact magnitude and duration of the event.

**Figure 5:**
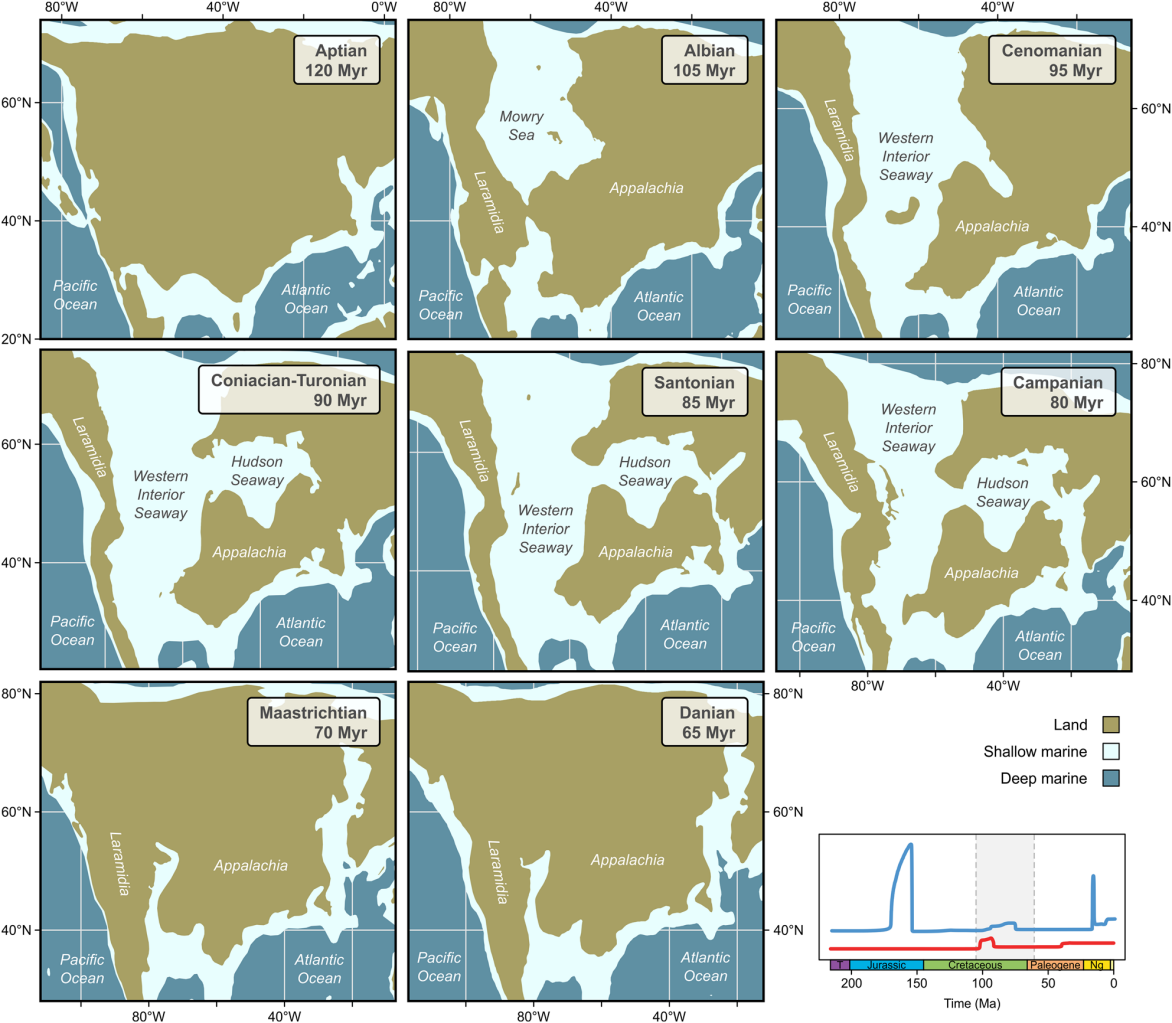
Paleogeography of North America during the mid-Cretaceous to early Paleocene, showing the major developments and phases of the Western Interior Seaway and adjacent regions. The graph on the bottom right indicates the temporal extent of the Western Interior Seaway and the corresponding shifts in the speciation (blue) and extinction (red) rates (see also Fig. 1). Map was generated in R v. 4.1.2 (https://www.r-project.org/) based on polygons from Ref.^27^. Region names follow Ref.^30^.

The mid-Cretaceous peak in the extinction rate coincides with the onset of the expansion of the Western Interior Seaway (WIS). This extensive marine incursion intermittently connected the Arctic Ocean with the Gulf of Mexico and the Atlantic Ocean from the late Early Cretaceous (late Albian) to the Paleocene (Danian/Selandian) (Fig. 5)^29,30,41–43^. We hypothesize that the progression of the marine WIS, following a period of extensive freshwater deposition in western North America during the Aptian to earliest Albian^29,30^, caused the extinction of numerous local freshwater faunas. In fact, the loss of this speciose record near the end of the Early Cretaceous is the first in a succession of WIS-influenced faunal turnovers. A second major loss of species occurred at the transition between the Early and Late Cretaceous (late Albian to middle Cenomanian). Most notable for that interval are the Bear River, Cloverly, and Kootenai Formations with faunas dominated by Amnicolidae, Hydrobiidae, Viviparidae, and Pleuroceridae^44–47^. Following an interval with few faunas (late Cenomanian–Santonian), an abundant continental biota developed in the latest Cretaceous (Campanian and Maastrichtian) with the progradation of freshwater environments^48,49^. The deposits of that time interval (e.g., Foremost, Judith River, St. Mary River, Laramie, Lance, and Hell Creek Formations) are rich in Pleuroceridae, Viviparidae, Physidae, Planorbidae, Hydrobiidae, Ampullariidae, Valvatidae, and other families^47,50,51^. The ongoing faunal turnovers are reflected in the speciation and extinction rates being elevated for millions of years (Fig. 1).

Despite the coincidence between the peak in extinction and the onset of the WIS, no correlation between extinction rate and continental fragmentation could be detected. This lack of correlation is likely a result of the constant extinction rate over most of the study interval. Generally, we would expect that major paleogeographic changes influence faunal developments due to their impact on the available ecosystems and hydrological connectivity, while smaller or gradual shifts in the continental landscape may be difficult to detect and can cause an overprinting (absent) long-term trend.

### The role of temperature in continental diversification

The results of our birth–death models with temperature allow for two main conclusions. First, regional temperature is a relevant control of diversification of freshwater biota on a continental scale. This finding confirms previous studies that demonstrated the influence of temperature variation on diversification rates across taxa, geographic areas, and stratigraphic intervals^2,3,12,21,22,25,52^. The positive correlations in our models (Fig. 2a) indicate that both speciation and extinction rates increase in warm periods. However, the trends seem to be strongly influenced by a few events in the Cenozoic. The major peak in speciation in the Neogene coincides with the onset of the Miocene Climate Optimum (MCO) c. 17 Myr ago (Fig. 1)^53^. Similarly, the rise in the extinction rate in the Paleogene matches the Middle Eocene Climate Optimum (MECO) c. 40 Myr ago (Fig. 1)^53^. Both events triggered shifts in the diversification and/or the migration of mammals, including hominoids^54,55^. Both MCO and MECO were also suggested to be responsible for extinction crises and migrations in European land snails^56^. Conversely, the shifts in speciation and extinction we found in the Mesozoic correlate with general cooling trends (Fig. 3).

Despite major climate changes over the study time interval (Fig. 3), prolonged periods are characterized by constant diversification rates (Fig. 1). This is probably the reason why a long-term association explains rate variation only marginally if at all better than a constant model (Fig. 2b), much like for the link between extinction rate and continental fragmentation. Only short-term and rapid climatic events seem to affect diversification of freshwater gastropods, while long-term climate change is apparently of little relevance. This pattern contrasts the recent finding that an interaction of long-term and short-term temperature changes affect speciation rates in marine Phanerozoic biota^57^.

Our result has an immediate implication for the current biodiversity crisis. The current pace of global climate change exceeds by far a natural rate^58^. North American freshwater gastropods are already under severe pressure through habitat destruction or alteration, pollution, and invasive species, amongst other factors^59^. Predicted future shifts in freshwater habitat suitability as a result of climate change across North America will likely increase stress on freshwater biota^60–62^. Our results imply that the rapid climate deterioration will come with massive long-term ramifications for diversification in North American freshwater gastropods^63^.

The second aspect of our results on temperature concerns the finding that global temperature is a poor proxy for deep-time diversification on a continental scale. Although studies have shown a significant relationship between continental diversification trends and global temperature^2^, our analyses indicate that regional estimates should be used instead^52^. Over extended geological time spans, tectonic displacement of continents across different latitudinal/longitudinal ranges involves shifts in regional climatic regimes. This is also the case for the North American continent during the Mesozoic and Cenozoic^27^. Consequently, we advise the use of regional temperature estimates based on raster climate models to assess the impact of temperature variation on diversification in continents or regional settings in general.

### Diversity dependence is of little relevance

Diversity dependence seems to have no influence on the diversification of North American freshwater gastropods on an extended temporal scale. There has been a long and intensive debate concerning the influence of diversity dependence and whether there is an upper limit to diversity in general^24,64,65^. Conceptually, diversity dependence refers to the concept that increasing diversity leads to increasing biotic interaction (e.g., competition for limited resources), which in turn causes a decline in the speciation rate and/or a rise in the extinction rate^11,65^. Numerous studies found support for diversity dependence across different species groups, time intervals, and geographic regions^2,4,15–17,65^. Among European fossil freshwater gastropods, diversity dependence was recently reported as the most important control on diversification over short geological timescales^18^.

The discrepancy among the results found by previous studies, also concerning the question if diversity dependence or upper limits to diversity exist at all, is likely owed to the different temporal, spatial, and taxonomic scales applied^65^. Our results indicate that diversity dependence is only of limited relevance to freshwater biota on a continental scale over extended geological time spans. In contrast, biotic factors are presumably more important at local and regional scales (e.g., in single drainage basins or biogeographic regions) and over shorter time spans^18,23^. The absence of diversity dependence of the extinction rate suggests that North American freshwater gastropod faunas were far from approaching equilibrium diversity.

### Limitations

The uneven sampling (Figs. S1, S3) and variable preservation over geological time and across the North American continent complicate reconstructions. Fossil data are derived primarily from the western plains and intramontane basins of the United States and Canada, while other parts of North America are rather poor in freshwater deposits and freshwater mollusks^66,67^ (Fig. S1). However, since the links to environmental changes concern globally relevant climatic events (MECO, MCO) and paleogeographic reorganizations concentrated in regions with rich faunas, we do not expect a strong bias in our reconstructions. Nonetheless, potential environmental events that may influence diversification in regions where no or few faunas are preserved are not covered by this study. This concerns, for example, large parts of eastern North America or the regions where marine deposition prevailed over prolonged periods (e.g., those covered by the Western Interior Seaway), which left limited space for freshwater ecosystems and their faunas to unfold. Similarly, certain time intervals lack data, such as the period covered by the Sundance Sea or parts of the Early Cretaceous (Fig. S3a). This results in wider credible intervals in speciation and extinction as well as preservation rates (Figs. 1, S2, S3b), which is why associated peaks or apparently constant rates should be interpreted with caution. Future investigations should attempt to incorporate spatial biases in the analyses^68–70^.

Finally, it is important to note that, in contrast to Europe, North America harbored only few long-lived lakes, in which species diversity and endemism tends to be high. Long-lived lakes typically produce extensive sedimentary deposits that have a higher chance of preserving mollusk faunas than their short-lived equivalents or rivers^71^. A prominent example is Late Jurassic Lake T’oo’dichi’, an alkaline–saline wetland–lake system that existed for approximately 2 Myr in the southern part of the Bighorn wetlands and deposited parts of the Morrison Formation^37,72^. A Cenozoic representative is the lake system in the Uinta–Piceance–Greater Green River Basin system, which contains lacustrine deposits that span several million years in the early Eocene^73^. Instead, North America’s fossil freshwater faunas are more commonly found in near-shore and riverine settings as well as short-lived lacustrine systems. Also, the poor preservation of shells in the many North American freshwater systems limits the actual known diversity^74,75^. The difference between North American and European freshwater faunas raises the interesting question whether the absence of long-lived lakes in North America could have depressed both speciation and extinction rates relative to the pattern in Europe, where the origin and demise of large ancient lakes greatly increased speciation and extinction^33,76,77^.

## Conclusion

Our results highlight the relevance of abiotic factors for diversification of continental freshwater biota and the timescales at which they act. Diversification rates of North American freshwater gastropod faunas are constant over long periods of time. Continental fragmentation—especially the changes in hydrological connectivity of freshwater ecosystems it entails—and regional temperature showed a significant long-term effect on the speciation rate, while variation in the extinction rate could not be explained by any factor. Diversity dependence showed no correlation at all. The apparent long-term influence of the abiotic factors, however, seems only a combined result of the impact of abiotic factors during selected periods or even short-term events. Notable peaks in the speciation and extinction rates coincide with major paleogeographic reorganizations in the Mesozoic, i.e., the formation of the Bighorn wetlands following the Sundance Sea (Late Jurassic) and the Western Interior Seaway (late Early Cretaceous to Paleocene), as well as with climatic events in the Cenozoic, i.e., the Middle Eocene Climate Optimum and the Miocene Climate Optimum. Especially the formation and retreat of the Mesozoic seaways coincided with extensive adjacent freshwater deposition, which likely provided impetus for the observed shifts in diversification. Considering the episodic nature of the climatic and paleogeographic changes coinciding with rate shifts, compared to long intervals without detectable shifts in diversification, statistical analyses based on long-term trends may fail to detect short-term influence. As such, our working hypothesis is only partly accepted—abiotic factors, and especially continental/hydrological fragmentation, are more important for diversification processes than biotic factors, but a significant long-term effect could not be supported. Future research may lead to the identification of specific time intervals or geographic regions to disentangle the diversification processes at different spatiotemporal scales.

## Material and methods

### Fossil dataset

North American freshwater mollusks have been the subject of a long research history dating back more than 170 years^44,78,79^. Especially during colonial westward expansion in the second half of the nineteenth century, numerous continental fossil faunas were described in the course of extensive geological surveys^50,80–83^. Since then, the diverse fossil faunas in the intramontane basins of the Rocky Mountains and beyond have sparked further research^42,45–49,75,84–93^. Ref.^44^ was the first to provide a comprehensive overview of the fossil record of the continental mollusks in the twentieth century. Later summaries dealt with specific time intervals^94^, geographic regions^47,74,95–97^ or major faunas^39^. Additionally, Ref.^98^ discussed the fossil record and diversity trends of several families in his review on global freshwater mollusk biogeography.

We compiled the fossil species occurrences of Late Triassic to Pleistocene North American freshwater gastropods from the literature^18^ (Fig. S1). For systematic classification (genus and family), rank (species or subspecies) and taxonomic status (valid or synonym), we followed the latest opinion as published in the literature. Likewise, the stratigraphic attribution of the fossil-bearing strata was taken from the most recent geological assessments to avoid a bias of the results from outdated chronostratigraphy. Species with uncertain identification, *nomina dubia*, *nomina nuda*, and *taxa inquirenda* were excluded from the analyses, while “good” taxa with invalid names (e.g., being junior homonyms pending revision) were included.

Contrasting other studies on long-term diversity or diversification trends that focus on the genus level^99–101^, we use the species as the basic unit for the analyses for two reasons. First, using genera in evolutionary studies has been shown to severely bias the results in some cases^102^. Second, the genus classification is difficult for many fossil freshwater gastropod species given the sometimes poor preservation (especially in Mesozoic strata) and the paucity of diagnostic characters. This typically leads to classification in overly broad (and often extant) genera that results in unrealistically long stratigraphic ranges.

The geographic delimitation of North America through geological time follows the present-day borders of countries and ranges from Alaska, Canada, and Greenland in the North to the Isthmus of Panama in the South. Excluded are Iceland and Svalbard, which are presently associated with Europe, but were connected to the North American continent at times^27^. Despite major paleogeographic changes affecting the connectivity of North America and adjacent continents since the Triassic^27^, assemblages known from coeval deposits of other continents bear little resemblance to the studied faunas^103^. Except for very few widespread species in the Pleistocene, which have a (nearly) Holarctic distribution [e.g., *Aplexa hypnorum* (Linnaeus, 1758)^104,105^], the North American fossil freshwater gastropod fauna is distinct. Alleged records of species originally described from North America in Asian fossil deposits^106^ are likely misidentifications and require revision (pers. obs. J.H.H., T.A.N., M.H.).

The final dataset contained 606 species and 3567 taxon occurrences in 1003 localities^107^.

### Diversification rate analyses

Diversification rates were inferred with the free software PyRate v. 3^108^ (https://github.com/dsilvestro/PyRate) following the protocol of Ref.^109^. To take age uncertainties into account, we ran the analyses for 100 randomized data sets, resampling the age of each species occurrence evenly within its fossil range. The algorithm jointly models fossil species occurrences as a result of speciation, extinction, and preservation, while accounting for sampling heterogeneity through geological time. Sampling is modeled as a time-variable Poisson process according to stratigraphic epochs. The Reversible Jump Markov Chain Monte Carlo (RJ-MCMC) algorithm was used to infer time-varying speciation and extinction rates, including the estimation of the number and temporal placement of rate shifts over time.

The basic analysis setting involved 50 million MCMC generations with a sampling frequency of 5000. Output files yielding a low effective sampling size (ESS < 100) were re-analyzed with 100 million MCMC generations but at the same sampling frequency to achieve convergence. ESSs were calculated with the package ‘coda’ v. 0.19-3^110^ for the statistical programming environment R v. 4.1.2^111^. In all cases, the initial 20% of the samples were discarded as burn-in. Rate plots were created at a temporal resolution of 0.1 Myr and are based on 100 randomly sampled generations per replicate.

### Potential controls on diversification

To test for a link between diversification rates and paleogeography, climate, and species diversity, we employed the univariate birth–death model implemented in the PyRate package^112^. As a measure for continental fragmentation we used the Shoreline Development Index (SDI). This index was originally introduced to quantify the outline complexity of lakes and describes the perimeter (P) deviation from a perfect circle with the same area (A) as the lake^113^:

$$SDI=\frac{P}{2\sqrt{A\pi }}$$

We reconstructed the area and perimeter of the North American continent through time based on latest paleogeographic reconstructions^27^. The study time interval (Late Triassic–Pleistocene) includes the opening of the northern Atlantic Ocean, when North America adjoined to large parts of South America, Africa, and Europe during the Late Triassic and Jurassic. Where seaways were absent, the border of North America was defined based on boundaries of tectonic plates and reconstructed country borders, as employed by the PALEOMAP project^114^. Boundary polygons were created in GPlates v. 2.2^115^. Using the R packages ‘raster’ v. 3.4-5^116^, ‘rgeos’ v. 0.5-5^117^, and ‘sf’ v. 0.9-7^118^, paleogeographic maps were cropped based on the boundary polygons and converted themselves to polygons. These were subsequently simplified using the function ‘gSimplify’ of the R package ‘rgeos’ with a numerical tolerance of 10 and afterwards smoothed using the package ‘smoothr’ v. 0.2.2^119^ and a Gaussian kernel regression with a smoothness factor of 3. Islands and holes in polygons greater than 5000 km^2^ were deleted subsequently with ‘smoothr’. The final polygons defining the North American continent at a given time slice were unified, and areas and perimeters were calculated with the package ‘geosphere’ v. 1.5-14^120^ based on a WGS84 ellipsoid (Fig. 3a,b).

The simplification and smoothing procedure hardly affects the area approximation but is particularly relevant for the reconstruction of comparable perimeters through time as past outlines are less certain than present-day ones. However, despite these efforts, the polygon for today’s North America is still much more accurate and thus has a higher outline complexity. To avoid any bias of the analyses from these differences in resolution, we omitted the recent time bin and ran the analyses (for all abiotic and biotic factors) for the time interval 5–215 Myr ago. In doing so, we also limited a potential pull-of-the-recent effect.

For temperature, we used both global and regional estimates to test for a variable influence of the spatial scale of observation. We focus here on regional temperature, considering that climate variation across the North American continent is more likely to directly influence diversification than global temperature^52^. Nonetheless, we also include global temperature as it is a commonly used measure in diversification studies. Using both factors allows testing for differences in the impact of regional and global climate.

Global average air temperature derived from the latest model of Ref.^28^ on a 1-Myr resolution (Fig. 3d). Regional temperature was reconstructed based on global temperature raster data on a 1° × 1° spatial resolution. These rasters derive from the PALEOMAP Project, which has produced paleogeographic maps at 5-Myr intervals^121^ and has assembled related temperature data based on the HadleyCM3 paleoclimate simulations^122^, which use estimates of the past concentration of atmospheric CO_2_^123^. It is a well-known observation that global climate models tend to produce cooler temperatures at higher latitudes, especially for hot house time intervals. In order to correct this bias, we adjusted the latitudinal temperature gradient using the Phanerozoic climate model^28^. This resulted in an average increase in temperature of c. + 5 °C at 60° latitude. North American average (“regional”) temperatures were calculated based on the intersection of the global temperature raster data and paleogeographic reconstructions^27^ with the R package ‘raster’ (Fig. 3d). The data for SDI, global, and regional temperature were centered and scaled by their standard deviations to facilitate comparison and interpolated to 0.1 Myr increments.

To account for age uncertainties of the fossil record, estimated times of origination and extinction for each species were extracted once for each of the 100 replicates (see previous chapter). The exponential correlation model was used for all three predictor variables and compared to a constant model, where rates are treated as invariable through time^112^. The unifactorial diversification models were ranked by their fit as quantified through marginal likelihoods *K*, which were inferred through thermodynamic integration in PyRate^108^. This method approximates the marginal likelihood of the data via ten MCMCs with increasingly altered acceptance ratio^124^, each running for 1,250,000 generations with a sampling frequency of 5000 and a burn-in of 10%. Output files yielding an ESS < 100 were re-analyzed with 2,500,000 or 3,000,000 MCMC generations but at the same sampling frequency to achieve convergence. Median Bayes factors (BF) comparing marginal likelihoods of alternative models across the 100 replicates were evaluated, whereas Δ2 log BF > 2 was considered as evidence for significant differences^125^. Correlations with biotic and abiotic factors were found significant if the 95% credible interval did not include zero^108^.

## Supplementary Information

Supplementary Figures.

Supplementary Table 1.

## Data Availability

The data that support the findings of this study are available in the supplementary material of this article. The dataset is available at 10.22029/jlupub-466.

## References

[1] Ricklefs RE (2010). Evolutionary diversification, coevolution between populations and their antagonists, and the filling of niche space. Proc. Natl. Acad. Sci. USA.

[2] Silvestro D, Antonelli A, Salamin N, Quental TB (2015). The role of clade competition in the diversification of North American canids. Proc. Natl. Acad. Sci. USA.

[3] Lehtonen S (2017). Environmentally driven extinction and opportunistic origination explain fern diversification patterns. Sci. Rep..

[4] Aguilée R, Gascuel F, Lambert A, Ferriere R (2018). Clade diversification dynamics and the biotic and abiotic controls of speciation and extinction rates. Nat. Commun..

[5] Wilke T (2020). Deep drilling reveals massive shifts in evolutionary dynamics after formation of ancient ecosystem. Sci. Adv..

[6] Bush AM, Payne JL (2021). Biotic and abiotic controls on the phanerozoic history of marine animal biodiversity. Annu. Rev. Ecol. Syst..

[7] Cantalapiedra JL (2021). The rise and fall of proboscidean ecological diversity. Nat. Ecol. Evol..

[8] Condamine FL, Guinot G, Benton MJ, Currie PJ (2021). Dinosaur biodiversity declined well before the asteroid impact, influenced by ecological and environmental pressures. Nat. Commun..

[9] Condamine FL, Rolland J, Morlon H (2013). Macroevolutionary perspectives to environmental change. Ecol. Lett..

[10] Condamine FL, Rolland J, Höhna S, Sperling FAH, Sanmartín I (2018). Testing the role of the red queen and court jester as drivers of the macroevolution of apollo butterflies. Syst. Biol..

[11] Rabosky DL (2013). Diversity-dependence, ecological speciation, and the role of competition in macroevolution. Annu. Rev. Ecol. Syst..

[12] Cantalapiedra JL (2014). Dietary innovations spurred the diversification of ruminants during the Caenozoic. Proc. R. Soc. B.

[13] Valente LM, Etienne RS, Phillimore AB (2014). The effects of island ontogeny on species diversity and phylogeny. Proc. R. Soc. B.

[14] Whittaker RJ, Fernández-Palacios JM, Matthews TJ, Borregaard MK, Triantis KA (2017). Island biogeography: Taking the long view of nature’s laboratories. Science.

[15] Quental TB, Marshall CR (2010). Diversity dynamics: Molecular phylogenies need the fossil record. Trends Ecol. Evol..

[16] Wiens JJ (2011). The causes of species richness patterns across space, time, and clades and the role of "ecological limits". Q. Rev. Biol..

[17] Pontarp M, Wiens JJ (2017). The origin of species richness patterns along environmental gradients: Uniting explanations based on time, diversification rate and carrying capacity. J. Biogeogr..

[18] Neubauer TA (2022). Drivers of diversification in freshwater gastropods vary over deep time. Proc. R. Soc. B.

[19] Ezard THG, Aze T, Pearson PN, Purvis A (2011). Interplay between changing climate and species' ecology drives macroevolutionary dynamics. Science.

[20] Ezard THG, Purvis A (2016). Environmental changes define ecological limits to species richness and reveal the mode of macroevolutionary competition. Ecol. Lett..

[21] Chaboureau A-C, Sepulchre P, Donnadieu Y, Franc A (2014). Tectonic-driven climate change and the diversification of angiosperms. Proc. Natl. Acad. Sci. USA.

[22] Davis KE, Hill J, Astrop TI, Wills MA (2016). Global cooling as a driver of diversification in a major marine clade. Nat. Commun..

[23] Xu L, Etienne RS (2018). Detecting local diversity-dependence in diversification. Evolution.

[24] Rabosky DL, Hurlbert AH (2015). Species richness at continental scales is dominated by ecological limits. Am. Nat..

[25] Condamine FL, Romieu J, Guinot G (2019). Climate cooling and clade competition likely drove the decline of lamniform sharks. Proc. Natl. Acad. Sci. USA.

[26] Kappes H, Tackenberg O, Haase P (2014). Differences in dispersal- and colonization-related traits between taxa from the freshwater and the terrestrial realm. Aquat. Ecol..

[27] Kocsis AT, Scotese CR (2021). Mapping paleocoastlines and continental flooding during the Phanerozoic. Earth-Sci. Rev..

[28] Scotese CR, Song H, Mills BJW, van der Meer DG (2021). Phanerozoic paleotemperatures: The earth’s changing climate during the last 540 million years. Earth-Sci. Rev..

[29] Miall AD, Catuneanu O, Vakarelov BK, Post R, Miall AD (2008). The Western Interior Basin. Sedimentary Basins of the World, Volume 5: The Sedimentary Basins of the United States and Canada.

[30] Slattery, J. S., Cobban, W. A., McKinney, K. C., Harries, P. J. & Sandness, A. L. Early cretaceous to paleocene paleogeography of the western interior seaway: the interaction of eustasy and tectonism. In *Wyoming Geological Association 68th Annual Field Conference Guidebook. Cretaceous Conference: Evolution and Revolution. Casper, Wyoming. June 1–4, 2013* (ed Bingle-Davis, M.) 22–60 (Wyoming Geological Association, 2015).

[31] Danise S, Holland SM (2017). Faunal response to sea-level and climate change in a short-lived seaway: Jurassic of the Western Interior, USA. Palaeontology.

[32] Danise S, Holland SM (2018). A sequence stratigraphic framework for the Middle to Late Jurassic of the Sundance Seaway, Wyoming: Implications for correlation, basin evolution, and climate change. J. Geol..

[33] Neubauer TA, Harzhauser M, Georgopoulou E, Kroh A, Mandic O (2015). Tectonics, climate, and the rise and demise of continental aquatic species richness hotspots. Proc. Natl. Acad. Sci. USA.

[34] Feng Y-J (2017). Phylogenomics reveals rapid, simultaneous diversification of three major clades of Gondwanan frogs at the Cretaceous-Paleogene boundary. Proc. Natl. Acad. Sci. USA.

[35] Minton R, McGregor B, Hayes D, Paight C, Inoue K (2017). Genetic structuring in the Pyramid *Elimia*, *Elimia potosiensis* (Gastropoda, Pleuroceridae), with implications for pleurocerid conservation. Zoosyst. Evol..

[36] Miura O, Urabe M, Mori H, Chiba S (2020). Ancient drainage networks mediated a large-scale genetic introgression in the East Asian freshwater snails. Ecol. Evol..

[37] Maidment SCR, Muxworthy A (2019). A chronostratigraphic framework for the Upper Jurassic Morrison Formation, western USA. J. Sediment. Res..

[38] Good SC (2004). Paleoenvironmental and paleoclimatic significance of freshwater bivalves in the Upper Jurassic Morrison Formation, Western Interior, USA. Sediment. Geol..

[39] Evanoff E, Good SC, Hanley JH (1998). An overview of the freshwater mollusks from the Morrison Formation (Upper Jurassic, Western Interior, USA). Mod. Geol..

[40] Wellborn GA, Langerhans RB (2015). Ecological opportunity and the adaptive diversification of lineages. Ecol. Evol..

[41] Hartman JH, Collins LS, Aubry M-P (1999). New interpretations of the Cannonball Formation (Paleocene)—North America's last interior sea. Geol. Soc. Am. Abstr. Prog..

[42] Hartman JH (2015). New viviparid gastropods from the end Cretaceous and early Paleogene of the Williston Basin, USA and Canada. Nautilus.

[43] Chang C, Liu L (2021). Investigating the formation of the Cretaceous Western Interior Seaway using landscape evolution simulations. GSA Bull..

[44] Henderson J (1935). Fossil non-marine Mollusca of North America. Geol. Soc. Spec. Pap..

[45] Yen T-C (1951). Fresh-water mollusks of cretaceous age from Montana and Wyoming. Part 1: A fluviatile fauna from the Kootenai formation near Harlowton, Montana. US Geol. Surv. Prof. Pap..

[46] Yen T-C (1954). Nonmarine mollusks of late cretaceous age from Wyoming, Utah and Colorado. Part 1: A fauna from western Wyoming. US Geol. Surv. Prof. Paper.

[47] Hartman JH (1998). The stratigraphy of Mesozoic and early Cenozoic nonmarine mollusks of Colorado. Proc. Denver Mus. Nat. Hist..

[48] Russell LS (1964). Cretaceous non-marine faunas of Northwestern North America. Life. Sci. Contrib. R. Ont. Mus..

[49] Hartman, J. H., Butler, R. D. & Bogan, A. Black Buttes Late Cretaceous continental and brackish mollusks: a critical geology stop on the Union Pacific Railroad, Sweetwater County, Wyoming. In *Wyoming Geological Association 68*th *Annual Field Conference Guidebook. Cretaceous Conference: Evolution and Revolution. Casper, Wyoming. June 1–4, 2013* (ed Bingle-Davis, M.) 88–125 (Wyoming Geological Association, 2015).

[50] Whiteaves JF (1885). Report on the invertebrata of the Laramie and Cretaceous rocks of the vicinity of the Bow and Belly Rivers and adjacent localities in the North-West Territory. Contrib. Can. Palaeont..

[51] Hartman JH, Kirkland JI (2002). Brackish and marine mollusks of the Hell Creek Formation of North Dakota: Evidence for a persisting Cretaceous seaway. Geol. Soc. Spec. Pap..

[52] Cantalapiedra JL, Domingo MS, Domingo L (2018). Multi-scale interplays of biotic and abiotic drivers shape mammalian sub-continental diversity over millions of years. Sci. Rep..

[53] Westerhold T (2020). An astronomically dated record of Earth’s climate and its predictability over the last 66 million years. Science.

[54] Begun DR, Güleç E, Geraads D (2003). Dispersal patterns of Eurasian hominoids: Implications from Turkey. Deinsea.

[55] Solé F (2015). A new large hyainailourine from the Bartonian of Europe and its bearings on the evolution and ecology of massive hyaenodonts (mammalia). PLoS ONE.

[56] Harzhauser M, Neubauer TA (2021). A review of the land snail faunas of the European Cenozoic—Composition, diversity and turnovers. Earth-Sci. Rev..

[57] Mathes GH, Kiessling W, Steinbauer MJ (2021). Deep-time climate legacies affect origination rates of marine genera. Proc. Natl. Acad. Sci. USA.

[58] Shukla PR (2019). Climate Change and Land: An IPCC Special Report on Climate Change, Desertification, Land Degradation, Sustainable Land Management, Food Security, and Greenhouse Gas Fluxes in Terrestrial Ecosystems.

[59] Johnson PD (2013). Conservation status of freshwater gastropods of Canada and the United States. Fisheries.

[60] Comte L, Buisson L, Daufresne M, Grenouillet G (2012). Climate-induced changes in the distribution of freshwater fish: Observed and predicted trends. Freshw. Biol..

[61] Hossain MA (2018). Assessing the vulnerability of freshwater crayfish to climate change. Divers. Distrib..

[62] Troia MJ, Kaz AL, Niemeyer JC, Giam X (2019). Species traits and reduced habitat suitability limit efficacy of climate change refugia in streams. Nat. Ecol. Evol..

[63] Hartman, J. H. & Bogan, A. The end of days—rethinking pre-Cretaceous-Paleogene boundary continental mussel nomenclature. In *9th North American Paleontological Convention (NAPC 2009), June 21–26, Cincinnati, Ohio, University of Cincinnati*. *Cincinnati Museum Center Scientific Contributions* Vol. 3, 141 (2009).

[64] Harmon LJ, Harrison S (2015). Species diversity is dynamic and unbounded at local and continental scales. Am. Nat..

[65] Marshall CR, Quental TB (2016). The uncertain role of diversity dependence in species diversification and the need to incorporate time-varying carrying capacities. Philos. Trans. R. Soc. B.

[66] Cobban WA, Reeside JB (1952). Correlation of the Cretaceous formations of the western interior of the United States. Bull. Geol. Soc. Am..

[67] Hartman, J. H. Upper Cretaceous Dakota Formation continental mollusks—resurrection of Western Interior Seaway eastern seaboard occurrences. In *Paleontology: Ancient Life in Deep Time: Temporal and Geographic Trends in Fossil Distributions. Abstracts with Programs (annual meeting, Baltimore, Maryland)* (eds Boag, T. & Manojlovic, M.), 3–9 (Geological Society of America, 2015).

[68] Close RA (2020). The apparent exponential radiation of Phanerozoic land vertebrates is an artefact of spatial sampling biases. Proc. R. Soc. B.

[69] Close RA, Benson RBJ, Saupe EE, Clapham ME, Butler RJ (2020). The spatial structure of Phanerozoic marine animal diversity. Science.

[70] Flannery-Sutherland JT, Silvestro D, Benton MJ (2022). Global diversity dynamics in the fossil record are regionally heterogeneous. Nat. Commun..

[71] Cohen AS (2012). Scientific drilling and biological evolution in ancient lakes: Lessons learned and recommendations for the future. Hydrobiologia.

[72] Demko, T. M., Nicoll, K., Beer, J. J., Hasiotis, S. T. & Park, L. E. Mesozoic lakes of the Colorado Plateau. Interior Western United States. *Geological Society of America Field Guide*, (eds Pederson, J. & Dehler, C. M.) Vol. **6** (2005).

[73] Smith ME, Carroll AR, Singer BS (2008). Synoptic reconstruction of a major ancient lake system: Eocene Green River Formation, western United States. GSA Bull..

[74] Hanley JH (1977). Lithostratigraphic relations, nonmarine Mollusca, and depositional environments of a portion of the Green River and Wasatch Formations south of the Rock Springs Uplift, Sweetwater County, Wyoming, with appendices of measured stratigraphic sections. Open-File Rep..

[75] Hartman, J. H. & Roth, B. Late Paleocene and early Eocene nonmarine molluscan faunal change in the Bighorn Basin, Northwestern Wyoming and South-Central Montana. In *Late Paleocene–Early Eocene Biotic and Climatic Events in the Marine and Terrestrial Records* (eds Aubrey, M.-P. *et al.*) 323–379 (Columbia University Press, 1998).

[76] Neubauer TA, Harzhauser M, Kroh A, Georgopoulou E, Mandic O (2015). A gastropod-based biogeographic scheme for the European Neogene freshwater systems. Earth-Sci. Rev..

[77] Neubauer TA, Georgopoulou E (2021). Extinction risk is linked to lifestyle in freshwater gastropods. Divers. Distrib..

[78] Hartman JH (2020). The importance of the museum in antebellum U.S. western territorial exploration: Understanding the relevance of collecting fossils and their conservation to solving long-standing geologic and paleontologic problems—Part 1. Earth Sci. Hist..

[79] Hartman JH (2021). The importance of the museum in antebellum U.S. western territorial exploration: Part 2. The roles of Hayden and Meek in a paradigm shift in geologic and paleontologic studies. Earth Sci. Hist..

[80] Evans J, Shumard BF (1856). Descriptions of new fossil species from the fresh water Tertiary Formation of Nebraska, Collected by the North Pacific Railroad Expedition, under Gov. J. J. Stevens. Proc. Acad. Nat. Sci. Philad..

[81] Meek FB, Hayden FV (1856). Descriptions of new fossil species of Mollusca collected by Dr. F. V. Hayden, in Nebraska Territory; together with a complete Catalogue of all the remains of Invertebrata hitherto described and identified from the Cretaceous and Tertiary Formations of that region. Proc. Acad. Nat. Sci. Philad..

[82] Meek, F. B. Preliminary paleontological report, consisting of lists and descriptions of fossils, with remarks on the ages of the rocks in which they were found, etc., etc. In *United States Geological and Geographical Survey of the Territories, Annual Report*, Vol. 6, 431–541 (1873).

[83] White, C. A. A review of the non-marine fossil Mollusca of North America. In *United States Geological Survey, Annual report (1881–1882)* Vol. 3, 403–550 (1883).

[84] Russell, L. S. Mollusca of the Paskapoo Formation in Alberta. In *Trans. R. Soc. Can., 3rd ser. Geol. Sci.* Vol. 20, 207–220 (1926).

[85] Russell LS (1931). Early tertiary Mollusca from Wyoming. Bull. Am. Paleontol..

[86] Pilsbry HA (1935). Mollusks of the fresh-water Pliocene beds of the Kettleman Hills and neighboring oil fields, California. Proc. Acad. Nat. Sci. Philad..

[87] Yen T-C (1952). Molluscan fauna of the Morrison formation. US Geol. Surv. Prof. Paper.

[88] Tozer ET (1956). Uppermost Cretaceous and Paleocene nonmarine molluscan faunas of western Alberta. Mem. Geol. Surv. Canada.

[89] Taylor DW (1960). Late Cenozoic molluscan faunas from the High Plains. US Geol. Surv. Prof. Paper.

[90] Pierce HG, Constenius KN (2001). Late Eocene-Oligocene nonmarine mollusks of the northern Kishenehn Basin, Montana and British Columbia. Ann. Carnegie Mus..

[91] Pierce HG, Constenius KN (2014). Terrestrial and aquatic Mollusks of the Eocene Kishenehn Formation, Middle Fork Flathead River, Montana. Ann. Carnegie Mus..

[92] Perrilliat MDC, Vega FJ, Espinosa B, Naranjo-Garcia E (2008). Late Cretaceous and Paleogene freshwater gastropods from northeastern Mexico. J. Paleont..

[93] Vega FJ, Naranjo-García E, Aguillón MC, Posada-Martínez D (2019). Additions to continental gastropods from the Upper Cretaceous and Paleocene of NE Mexico. Bol. Soc. Geol. Mex..

[94] Taylor DW (1966). Summary of North American blancan nonmarine mollusks. Malacologia.

[95] Hartman JH (1981). Early tertiary nonmarine mollusca of New Mexico: A review. GSA Bull..

[96] La Rocque A (1960). The molluscan faunas of the Flagstaff Formation of central Utah. Geol. Soc. Am. Mem..

[97] La Rocque A (1963). Late Cenozoic non-marine molluscan associations in eastern North America. Sterkiana.

[98] Taylor DW (1988). Aspects of freshwater mollusc ecological biogeography. Palaeogeogr. Palaeoclimatol. Palaeoecol..

[99] Alroy J (2008). Phanerozoic trends in the global diversity of marine invertebrates. Science.

[100] Silvestro D, Cascales-Miñana B, Bacon CD, Antonelli A (2015). Revisiting the origin and diversification of vascular plants through a comprehensive Bayesian analysis of the fossil record. New Phytol..

[101] Payne JL, Bush AM, Heim NA, Knope ML, McCauly DJ (2016). Ecological selectivity of the emerging mass extinction in the oceans. Science.

[102] Hendricks JR, Saupe EE, Myers CE, Hermsen EJ, Allmon WD (2014). The generification of the fossil record. Paleobiology.

[103] Neubauer TA (2021). Current extinction rate in European freshwater gastropods greatly exceeds that of the late Cretaceous mass extinction. Commun. Earth Environ..

[104] La Rocque, A. Pleistocene Mollusca of Ohio. *State of Ohio*, *Department of Natural Resources*, *Division of Geological Survey*, *Bulletin* Vol. 62, xv-xxiv, 357–553 (1968).

[105] Welter-Schultes FW (2012). European Non-marine Molluscs, A Guide for Species Identification.

[106] Pan H, Zhu X (2007). Early Cretaceous non-marine gastropods from the Xiazhuang Formation in North China. Cretac. Res..

[107] Neubauer, T. A. *et al.**Data for “Climate, paleogeography, and the diversification of North American freshwater gastropods”*10.22029/jlupub-466 (2022).

[108] Silvestro D, Salamin N, Schnitzler J (2014). PyRate: A new program to estimate speciation and extinction rates from incomplete fossil data. Methods Ecol. Evol..

[109] Silvestro D, Salamin N, Antonelli A, Meyer X (2019). Improved estimation of macroevolutionary rates from fossil data using a Bayesian framework. Paleobiology.

[110] Plummer, M. et al. coda: Output Analysis and Diagnostics for MCMC. R package version 0.19-3. https://cran.r-project.org/web/packages/coda/index.html (2019).

[111] R Core Team. R: A language and environment for statistical computing. Version 4.1.2. https://www.r-project.org/ (R Foundation for Statistical Computing, 2021).

[112] Silvestro D, Schnitzler J, Hoorn C, Perrigo A, Antonelli A (2018). Inferring macroevolutionary dynamics in mountain systems from fossils. Mountains, Climate and Biodiversity.

[113] Cole GA, Weihe PE (2016). Textbook of Limnology.

[114] Scotese, C. R. & Wright, N. PALEOMAP Paleodigital Elevation Models (PaleoDEMS) for the Phanerozoic. PALEOMAP Project, Evanston, IL. https://www.earthbyte.org/paleodem-resource-scotese-and-wright-2018/ (2018).

[115] Müller RD (2018). GPlates: Building a virtual earth through deep time. Geochem. Geophys..

[116] Hijmans, R. J. *et al.* raster: Geographic Data Analysis and Modeling. R package version 3.4-5. https://rspatial.org/raster/ (2020).

[117] Bivand, R. *et al.* rgeos: Interface to Geometry Engine—Open Source ('GEOS'). R package version 0.5-5. https://cran.r-project.org//rgeos/index.html (2020).

[118] Pebesma E (2018). Simple features for R: Standardized support for spatial vector data. R J..

[119] Strimas-Mackey, M. smoothr: Smooth and Tidy Spatial Features. R package version 0.2.2. http://CRAN.R-project.org/package=smoothr (2021).

[120] Hijmans, R. J., Karney, C., Williams, E. & Vennes, C. geosphere: Spherical Trigonometry. R package version 1.5-14. http://CRAN.R-project.org/package=geosphere (2021).

[121] Scotese CR (2021). an atlas of paleogeographic maps: The seas come in and the seas go out. Annu. Rev. Earth Planet. Sci..

[122] Valdes PJ, Scotese CR, Lunt DJ (2021). Deep ocean temperatures through time. Clim. Past.

[123] Foster GL, Royer DL, Lunt DJ (2017). Future climate forcing potentially without precedent in the last 420 million years. Nat. Commun..

[124] Lartillot N, Philippe H (2006). Computing Bayes factors using thermodynamic integration. Syst. Biol..

[125] Kass RE, Raftery AE (1995). Bayes factors. J. Am. Stat. Assoc..

