# Supplementary material for: Short-term paleogeographic reorganizations and climate events shaped diversification of North American freshwater gastropods over deep time
Source: Sci Rep. 2022 Sep 16;12:15572. doi: 10.1038/s41598-022-19759-4 (PMC9481594; doi:10.1038/s41598-022-19759-4)
Supplement: Supplementary file 1 — Supplementary Figures. [file 41598_2022_19759_MOESM1_ESM.docx]

**Short-term paleogeographic reorganizations and climate events shaped diversification of North American freshwater gastropods over deep time**

Supplementary Information

**Thomas A. Neubauer^1,2,3^, Mathias Harzhauser^4^, Joseph H. Hartman^5^, Daniele Silvestro^6,7^, Christopher R. Scotese^8^, Alexander Czaja^9^, Geerat J. Vermeij^10^ & Thomas Wilke^1^**

^1^Department of Animal Ecology and Systematics, Justus Liebig University, 35392 Giessen, Germany

^2^SNSB – Bavarian State Collection for Paleontology and Geology, 80333 Munich, Germany

^3^Naturalis Biodiversity Center, 2333 CR Leiden, The Netherlands

^4^Geological-Paleontological Department, Natural History Museum Vienna, 1010 Vienna, Austria

^5^University of North Dakota, Harold Hamm School of Geology and Geological Engineering, Grand Forks, ND 58202, USA

^6^Department of Biology, University of Fribourg, 1700 Fribourg, Switzerland

^7^Gothenburg Global Biodiversity Centre, University of Gothenburg, 413 19 Gothenburg, Sweden

^8^Department of Earth and Planetary Sciences, Northwestern University, Evanston, IL 60208, USA

^9^Facultad de Ciencias Biológicas, Universidad Juárez del Estado de Durango, Fraccionamiento Filadelfia, 35010 Gómez Palacio, Durango, Mexico

^10^Department of Earth and Planetary Science, University of California, Davis, CA, USA

Correspondence: Thomas A. Neubauer, SNSB – Bavarian State Collection for Paleontology and Geology, Richard-Wagner-Straße 10, 80333 Munich, Germany. E-mail: neubauer@snsb.de

This document contains Supplementary Figures S1–3

Supplementary figures


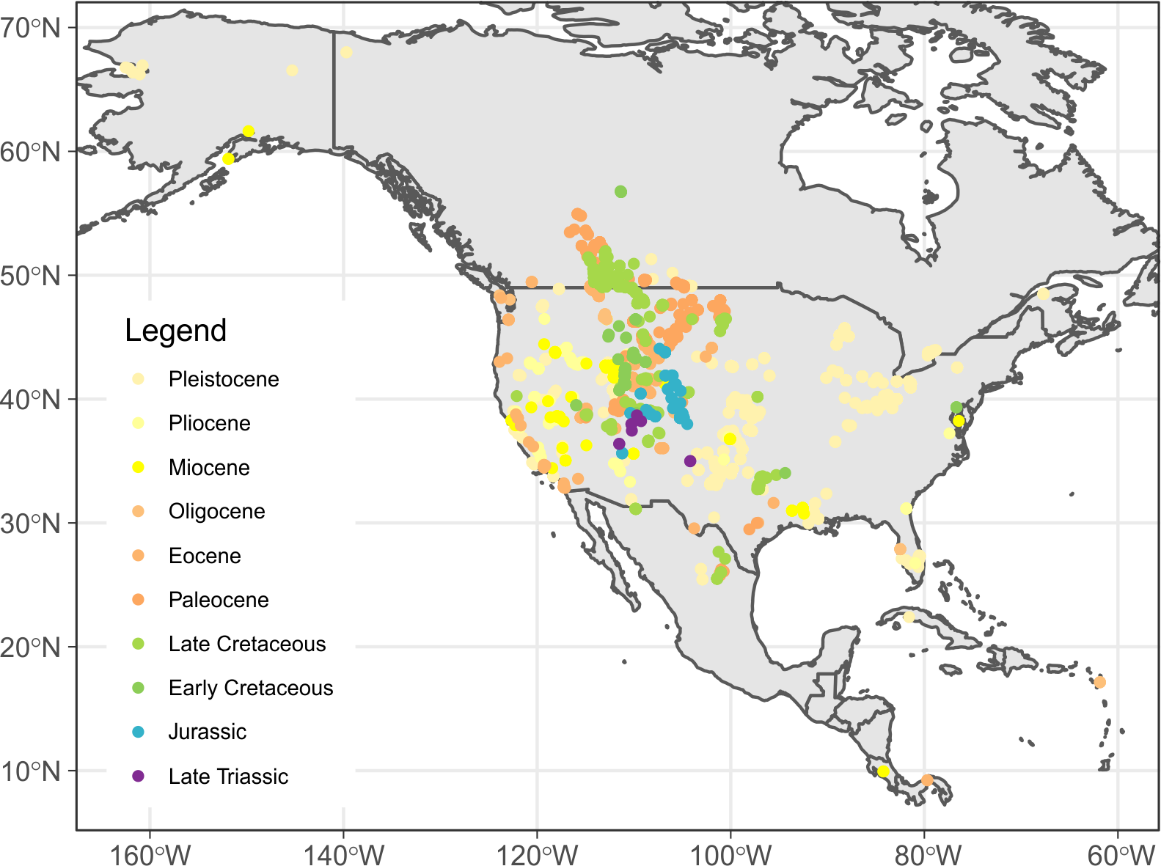


**Figure S1.** Map of North American localities yielding fossil freshwater gastropods, binned into stratigraphic epochs. Because of the comparably low number of records for Jurassic epochs, localities were combined into a single bin. Colors correspond to the International Chronostratigraphy Chart (https://stratigraphy.org/chart). Map was generated in R v. 4.1.2 (https://www.r-project.org/).


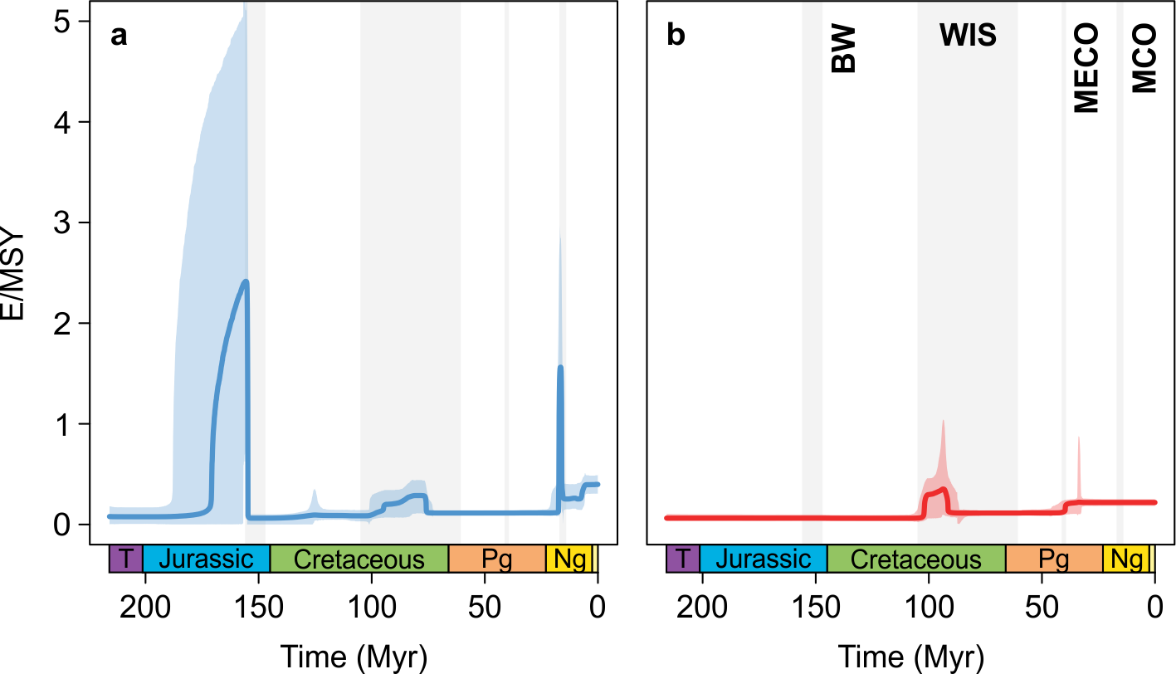


**Figure S2.** Speciation (a) and extinction (b) rates of North American freshwater gastropods from the Late Triassic to the Pleistocene (c. 214.0–0.0117 Myr). Shown are the mean rates and the 95% highest posterior density quantifying the uncertainty in rates. Time intervals with major seas/wetlands and climatic events referred to in the main paper are marked by gray bars. See Fig. 1 in main paper for truncated version. BW, Bighorn wetlands; E/MSY, events per million species years; MECO, Middle Eocene Climate Optimum; MCO, Miocene Climate Optimum; WIS, Western Interior Seaway.


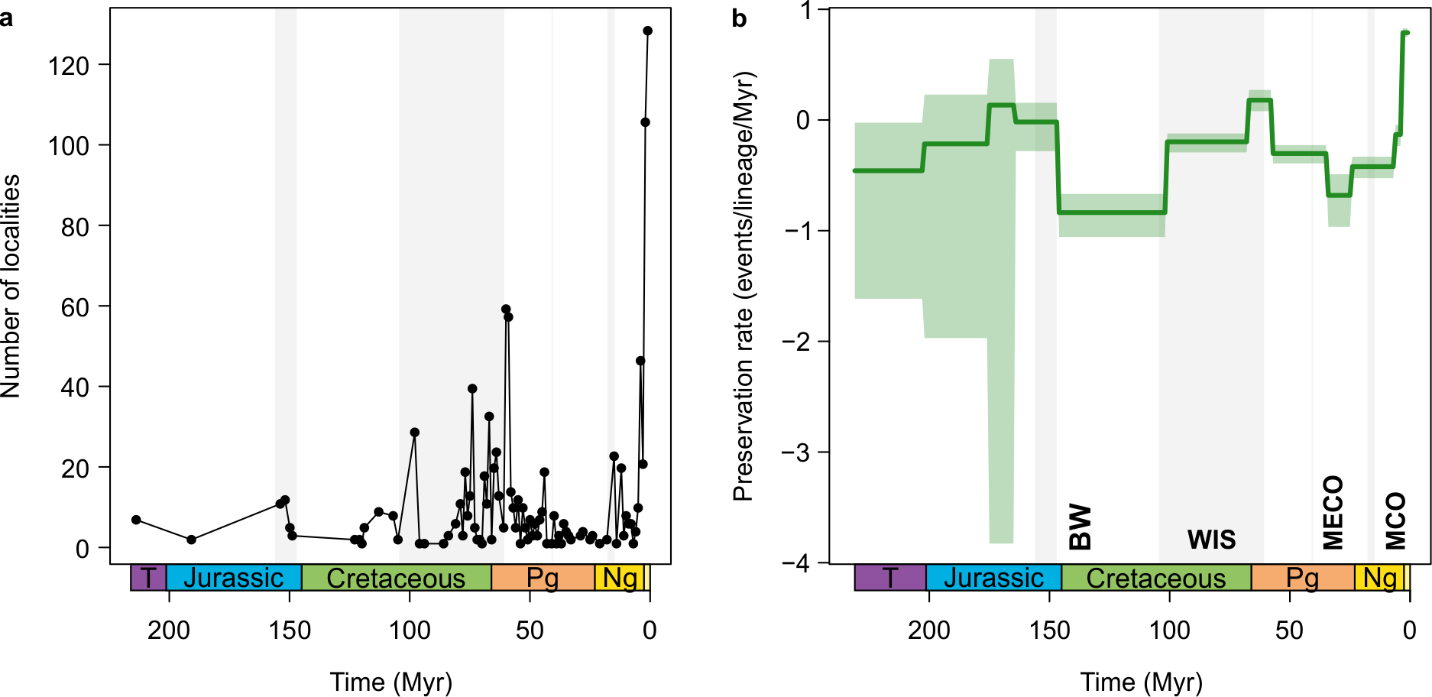


**Figure S3.** Sampling and preservation. (a) Number of fossil-bearing localities per 1-Myr bin. (b) Preservation rate (log10-transformed) estimated through PyRate v. 3, based on 100 replicates. Shown are the mean rates and the 95% highest posterior density quantifying the uncertainty in rates. Time intervals with major seas/wetlands and climatic events referred to in the main paper are marked by gray bars. BW, Bighorn wetlands; MECO, Middle Eocene Climate Optimum; MCO, Miocene Climate Optimum; WIS, Western Interior Seaway.
